# Analog Integrated Current Drivers for Bioimpedance Applications: A Review

**DOI:** 10.3390/s19040756

**Published:** 2019-02-13

**Authors:** Nazanin Neshatvar, Peter Langlois, Richard Bayford, Andreas Demosthenous

**Affiliations:** Department of Electronic and Electrical Engineering, University College London, Torrington Place, London WC1E 7JE, UK; p.langlois@ucl.ac.uk (P.L.); r.bayford@ucl.ac.uk (R.B.); a.demosthenous@ucl.ac.uk (A.D.)

**Keywords:** bioimpedance measurement, current driver, integrated circuit, linear feedback, nonlinear feedback, transconductance

## Abstract

An important component in bioimpedance measurements is the current driver, which can operate over a wide range of impedance and frequency. This paper provides a review of integrated circuit analog current drivers which have been developed in the last 10 years. Important features for current drivers are high output impedance, low phase delay, and low harmonic distortion. In this paper, the analog current drivers are grouped into two categories based on open loop or closed loop designs. The characteristics of each design are identified.

## 1. Introduction

Bioimpedance measurement is defined as the study of biological tissue/cell in response to an alternating electric field, which can cover a frequency range from tens of Hz to several MHz [1,2]. The electrical properties of tissue/cell (conductive and dielectric properties) are characterized by frequency variable complex electrical bioimpedance providing important information about the tissue/cell physiology and pathology. 

In order to measure the bioimpedance, a current stimulus is required to be provided through a set of electrodes and measuring the corresponding potential via the same, or another, pair of electrodes. Depending on whether to measure the real and imaginary components of the impedance, as in electrical impedance spectroscopy (EIS), or to image the impedance, as in electrical impedance tomography (EIT), a synchronous detection method [3], or finite element algorithms [4], can be utilized respectively.

To provide an accurate impedance measurement, the current injected into the tissue should have a constant and accurate amplitude throughout the required band of frequency. This imposes tight specifications on the design of the current driver. A current driver should have high output impedance with respect to load, so that all the current gets transferred to the load, and not shunted away. For example, an output impedance of 1 MΩ for a load of 1 kΩ will provide 0.1% accuracy of the output current. As the impedance is presented as real and imaginary or magnitude and phase values, it is of prime importance to have the minimum phase error at the output of the current driver. This is due to the fact that any phase variation at the output of the front end should be only due to the tissue under test, otherwise the phase errors between the driver and the measured potential cannot be distinguished from each other. It is appreciable to perform the impedance measurement up to 10 MHz range, as it can provide an insight to the nucleus of the cell [1]. 

This paper provides a review on multiple integrated current drivers, used mostly in EIS and EIT applications. They are configured in two groups of open loop and closed loop drivers. In each system, the transconductance and output impedance has been analyzed. The measurement results, in terms of current amplitude, bandwidth, and output phase error, are discussed. A brief analysis of advantages and limitations of each concludes each design.

## 2. Current Driver Designs

### 2.1. Open Loop Integrated Current Drivers

**Figure 1:**
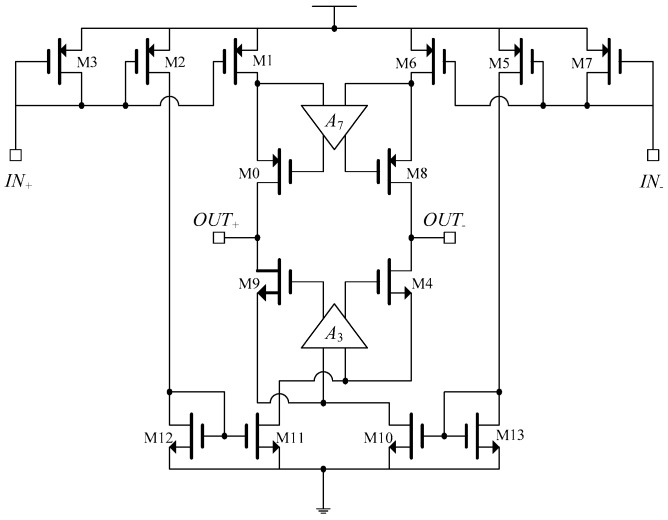
Integrated current driver in H-bridge configuration [5].

In [5], an integrated current driver was introduced using standard 0.18 μm CMOS technology. The schematic is shown in Figure 1.

This fully differential current driver employed four current sources in an H-bridge configuration. The two current sources, M1 and M10, are active during the positive half cycle of the sinusoidal reference input, while current sources, M6 and M11, are active in the negative half cycle [5]. Therefore, the current can flow in both directions through the load. Cascode transistor configuration with differential gain-boosting op-amps (*A*3, *A*7) were used to increase the output impedance of the current drivers. 

Due to symmetry of the system, the output impedance can be approximated by:

(1) $$Z_{o}=g_{mo}r_{o0}r_{o1}\left(A_{7}+1\right)\|g_{m9}r_{o9}r_{o10}\left(A_{3}+1\right)$$

where *g_mi_* is the transconductance and *r_oi_* is the drain to source resistance of the *i_th_* transistor. Simulations yield a high output impedance of approximately 10.2 MΩ at 1 MHz and a THD of 1% at 10 MHz for an output current of 107 μA_p-p_ [5]. The system operates in open loop configuration, and the output current accuracy is directly affected by internal parameters of the system. Since in an open loop system the gain variation is large, even a very small input can saturate the system. No information regarding the phase error is provided.

In this system, the output current was sensitive to the mismatch of the current source transistors. In order to have proper settling behavior for the system, the unity gain frequency of *A*_3_ and *A*_7_ should be less than the second pole of the main current driver [6]. Therefore, large transistor ratios (W/L) for the current source were necessary to increase the overall output capacitance of the current source, with respect to the *A*_3_ and *A*_7_ output capacitors.

As the frequency was increased, the open loop gain started to roll off at the –3dB of the system. Limited bandwidth of the current driver is an important factor in the reduction of output impedance at high frequencies. This is due to the fact that, as the frequency increases, the effects of the parasitic capacitors at the output of the current driver dominate. This results in a phase delay between the input and output, which can cause errors in impedance measurements.

**Figure 2:**
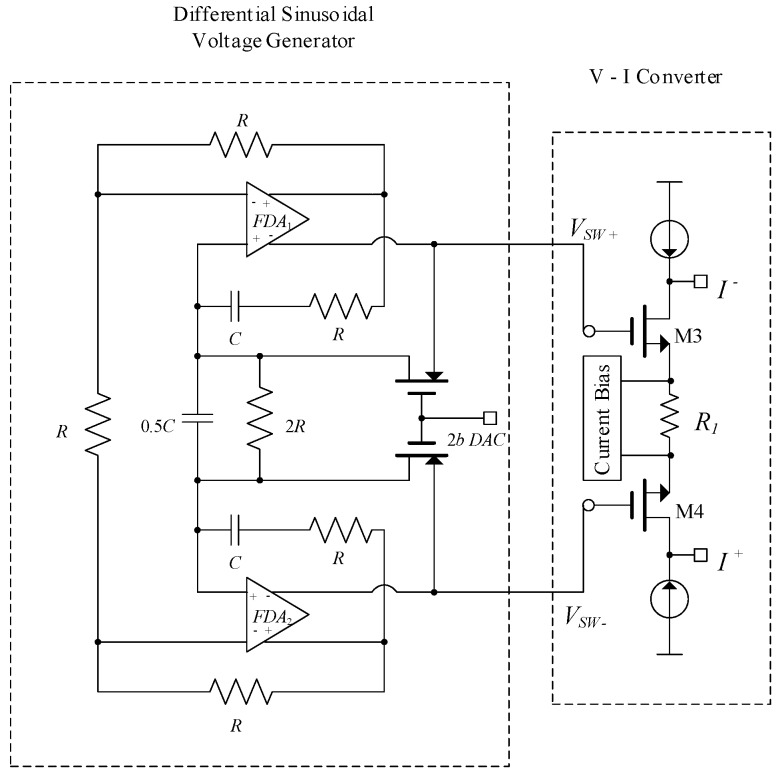
Current driver [7].

The current driver proposed in [7] is shown in Figure 2 and was realized in a 0.18 μm standard CMOS technology. It is composed of two fully differential amplifiers (FDA1 and FDA2) with an *RC* frequency selective network that produces an input sinusoidal voltage, followed by a VCCS, formed by transistors M3 and M4 and resistor *R*_1_ [7]. The FDA is a two-stage folded cascode with source followers at the output stage in order to achieve low output impedance to effectively drive the frequency selective *RC* bridge network [7]. The *RC* frequency selective network generated a 90 kHz balanced sinusoidal voltage signal *V_SW_^+^* and *V_SW_^−^*. The 2 bit-DAC output adjusts the gate voltages of transistors M3 and M4 that results in variable sinusoidal voltage of 0.22 V_p-p_ to 0.78 V_p-p_ [7]. 

The output current is defined as:

(2) $$I_{out}=I^{+}-I^{-}=\frac{V_{SW}{}^{+}-V_{SW}{}^{-}}{R_{1}}$$

The output impedance of the V/I converter is given by:

(3) $$Z_{o}=\frac{1}{g_{m3}}+\frac{1}{g_{m4}}+R_{1}$$

where *g_mi_* is the transconductance of transistor M_i_. Based on Equation (2), the output current is inversely proportional to *R*_1_. However, the absolute value of *R*_1_ can vary up to 20% which leads to inaccurate current measurements unless it is laser trimmed. According to Equation (3), a large output impedance can be achieved for small transconductance of transistors M3 and M4, or large *R*_1_. 

The system operated at 90 kHz and generated four-step controllable balanced sinusoidal currents of 100 µA_p-p_–350 µA_p-p._ The current driver operates in open loop configuration, and the output current is heavily dependent on internal parameters of the system, including the variation of the two FDA*s* that generate *V_SW_^+^* and *V_SW_^−^*. Moreover, the 2 bit-DAC limits the output current to four values only. Any offset generated by DAC and FDA will alter *V_SW_^+^* and *V_SW_^−^* and *I^−^* and *I^+^* respectively. Measured results verify a variation of 1% in the output current for loads less than 5.6 kΩ at 90 kHz [7]. Thus, the output impedance can be realized to be in excess of 560 kΩ at 90 kHz. Additionally, THD of the output current is stated to be less than 1% for 250 μA_p-p_ measured at 90 kHz [7]. The overall power consumption is 2 mW. 

An analogous architecture was adopted by the same group in another study [8], where instead of a DAC, an adaptive gain control was utilized to stabilize the output voltage swing of FDA. The oscillation frequency can be controlled by tunable resistor *R*. The operational frequency range of the driver was from 10 to 76 kHz. Different values of current were achieved through variable transconductance gain (*1/R*_1_). The voltage to current (V–I) converter translated the differential sinusoidal voltage from FDAs into balanced currents of 80, 200, and 400 μA via a variable transconductance gain of 1/*R*_1_ = 200 μA/V, 500 μA/V and 1 mA/V, respectively. The same equations for output current and output impedance as the previous current driver applies here. A THD of less than 0.2% was achieved for 200 μA_p-p_ [8]. 

**Figure 3:**
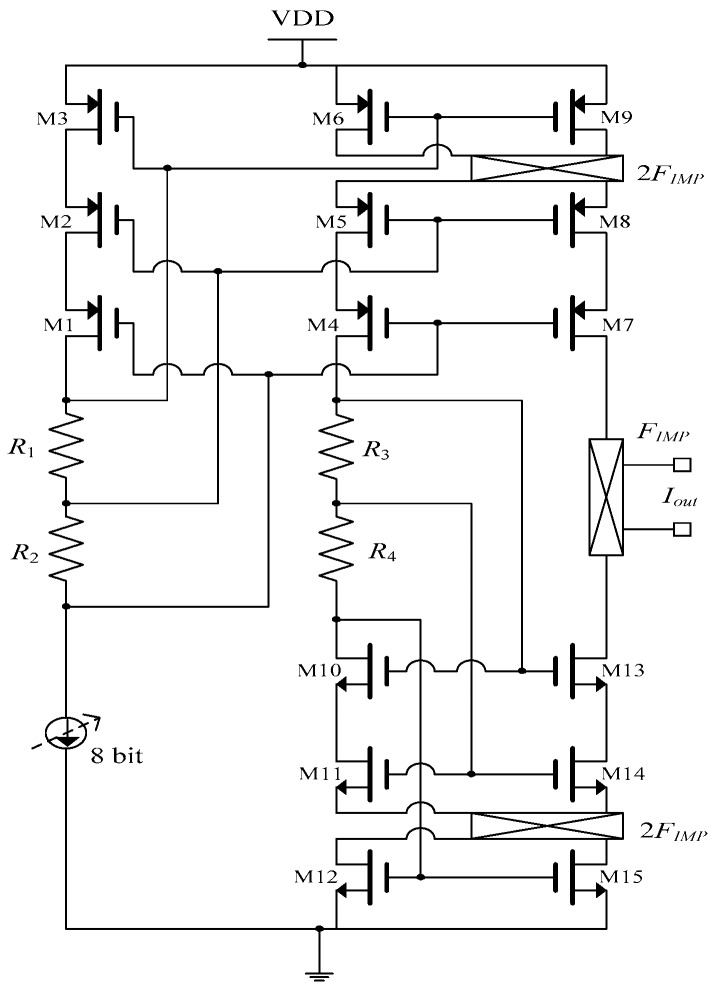
Circuit schematic of triple cascode current driver [9].

The current driver in [9] generates square currents up to 169 µA, and was fabricated in 0.13 µm technology with a supply voltage of 1.2 V. The amplitude of the excitation was set by an 8-bit current DAC, which is programmed from 0 to 169 µA in 256 steps. The schematic of this triple cascode current driver is shown in Figure 3.

A triple cascode architecture was used to achieve high output impedance. The biasing of cascodes was implemented via resistor division. Two chopper switches were used to average the mismatches among the cascode mirrors [9]. The switch at the output is to set the frequency of the alternating current. The output impedance of the current driver is defined as:

(4) $$Z_{o}=g_{m7}r_{o7}r_{o8}r_{o9}\|g_{m13}r_{o13}r_{o14}r_{o15}$$

In this architecture, the offset resulting from the mismatch of the cascode current mirrors is eliminated by use of choppers. In processes with small supply voltages, cascodes can be a limiting factor in the output swing. In this system, although the presence of triple cascode increases the output impedance, it severely limits the output current amplitudes, as each cascode consumes at least an effective gate driving voltage. Any transistor in the signal path translates into a pole in the transfer function. Achieving a high phase margin is not an easy task [6]. The on-chip resistors are prone to mismatch. Using them as a biasing technique to drive the cascodes may not be an optimum option, as the variation from fabrication may cause some transistors to go in to the triode region and reduce the output impedance of the driver. When high frequency operation is considered, all the parasitic capacitors from triple cascode, in addition to stray capacitors, will result in phase imbalance between the input and output.

**Figure 4:**
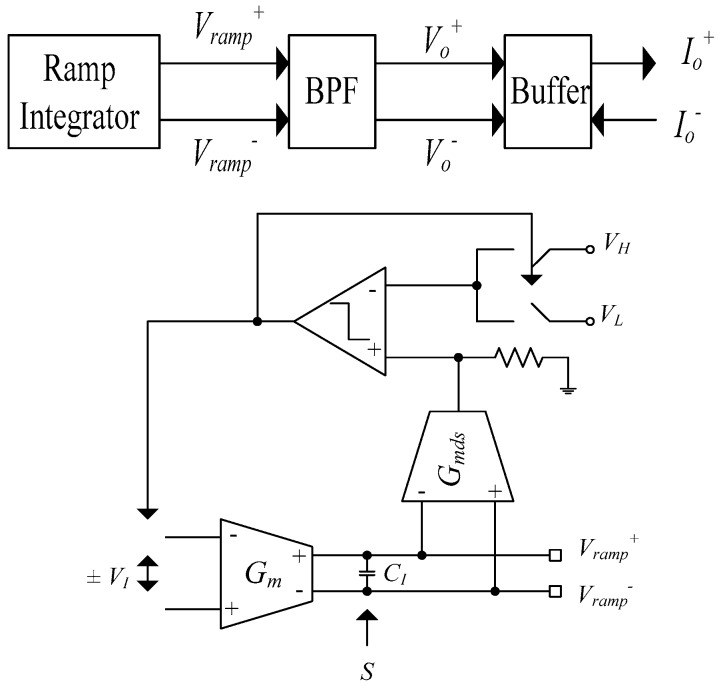
Block diagram of current driver and ramp generator [10].

A sinusoidal current driver based on ramp integrator and fabricated in 0.8 μm process with 3 V supply was discussed in [10]. The block diagram of the current driver is illustrated in Figure 4. The output is fed to a voltage to current buffer that is used to define the limits of the current amplitude and to reduce the loading effects. The system works at three distinct frequencies of 1, 8, and 16 kHz through a 2-bit digital control capacitor bank denoted by letter S. The maximum output current is 7 μA_p_. The oscillation frequency is defined as [10]

(5) $$f_{osc}=\frac{g_{m}V_{I}}{2\left(V_{H}-V_{L}\right)C_{I}}$$

where *V_I_* is a constant reference voltage generated on-chip, *C_I_* is the integrator capacitor, *g_m_* is the transconductance of the OTA, and *V_H_−V_L_* is the allowable voltage swing for the integrator output [10].

A *G_m_*–*C* ramp integrator, along with a second order band pass filter, was used to produce sinusoidal output voltages. 

A major drawback of this system is that it performs in open loop configuration. Hence, even a small input voltage can saturate the system. Moreover, the output current is directly affected by internal parameters of the system. The output impedance is defined by the output impedance of the buffer. Since the operating frequency is quite low, the capacitor effects of the limited bandwidth OTA in buffer are not a major issue. Good matching between the oscillation frequency of the ramp generator and band pass filter is a major task. As was mentioned in [10], as the frequency increased, the amplitude of the current decreased, which resulted in inaccurate current measurements. No information about measured THD and output impedance is provided. A high Q band pass filter is necessary for better synchronization and a higher order or a smaller bandwidth is likely required to suppress the even harmonics from the ramp generator to an acceptable level, in order to have a clean sinusoidal input to the buffer.

**Figure 5:**
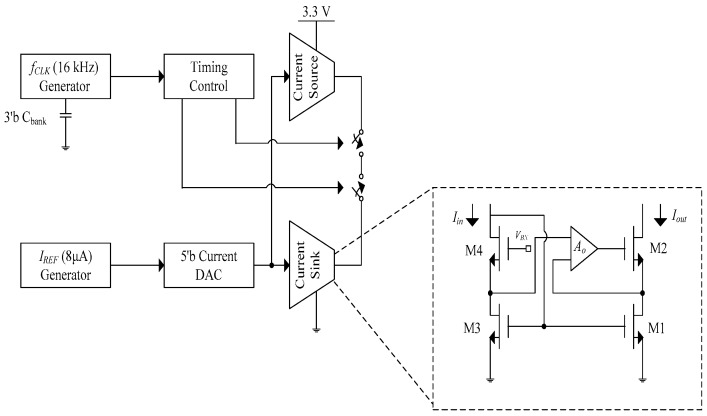
Block diagram of the current driver [11].

The current driver in [11] provides a current source and current sink from a 3.3 V supply. The schematic of the current driver is shown in Figure 5. The amplitude of the current is determined by a 5-bit current DAC, which can be programmed from 16 to 512 μA. A timing control block generates different frequencies in the range of DC–500 Hz from a nominal 16 kHz clock that can be calibrated through a 3-bit capacitor bank [11]. The output impedance of the system is:

(6) $$R_{out}=A_{o}g_{m2}r_{o2}r_{o1}$$

where *A_o_* is the open loop gain of feedback amplifier, *g_m_*_2_ is the transconductance of M2, and *r_o_*_1_ and *r_o_*_2_ are the output resistance of transistor M1 and M2, respectively. The measured output impedance is 1.8 GΩ. With a load of 5 kΩ, a current amplitude of 512 μA was observed. The same principle for the open loop gain systems applies here. Moreover, the current amplitude and frequency of operation are dictated by DAC and capacitor bank, respectively. Any offset from the DAC will alter the current accuracy.

**Figure 6:**
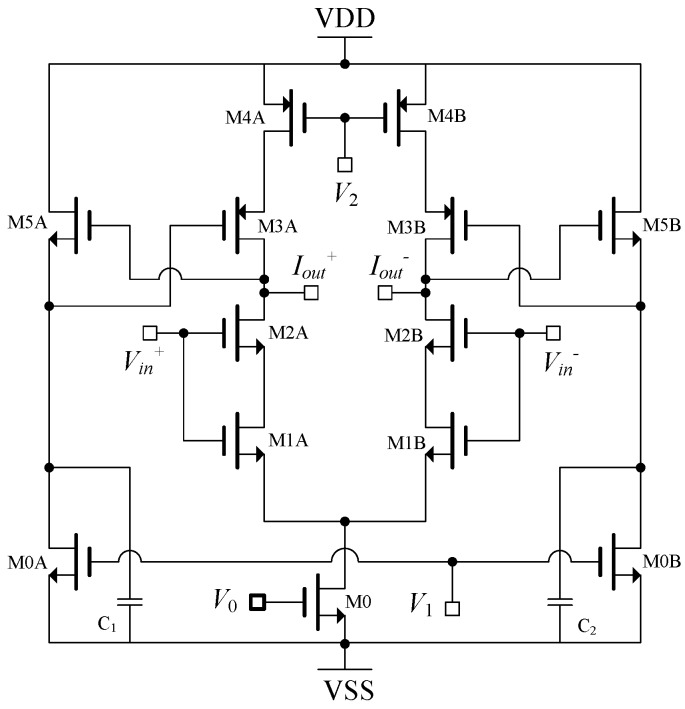
Open loop wideband current driver [12].

The current driver shown in Figure 6 can be utilized as a driver for EIT systems. Transistors M1A, M1B, M2A, and M2B form the voltage to current converter, and transistors M3A, M3B, M4A, M4B, M5A, and M5B form an active inductive load [12]. Transistors in active load configuration are categorized as M3A and M4A to form cascode load, and M5A and M0A to form a source follower to provide the feedback control of the gate voltage transistor M3A [12].

The high output impedance was achieved through the active load design by inserting a zero at low frequencies rather than using cascode stages. The transconductance of the current driver circuit is:

(7) $$G_{m}=\frac{g_{m}}{1+g_{m}R}$$

where *g_m_* is the small signal transconductance of input transistors M2A–B, which are linearized by source degeneration triode transistors formed by M1A–B and represented by R.

It is important to note that the transconductance of the current driver is directly proportional to *g_m_* of the transistors M2A–B, which is a design parameter for input transistors. Although the use of source degeneration is to linearize the transconductance, due to the small value of *g_m_R*, proper accuracy cannot be achieved. Even with the assumption of a large *g_m_R*, the transconductance will still be 1/*R*, which is a function of the drain current in the transistor.

The output impedance of the circuit is [12]

(8) $$Z_{o}=\frac{r_{o4}\left(1+j\omega \frac{C}{g_{m5}}\right)}{1+j\omega \frac{C}{g_{m2}r_{o2}g_{m5}}}$$

As a result, the output impedance at low frequencies is equal to *r_o4_*, which is the small signal output resistance of transistor M4. At high frequencies this can be varied by the location of zero and pole, which are [13]:

(9) $$\omega_{z}=\frac{g_{m5}}{C}$$

(10) $$\omega_{p}=\frac{g_{m5}g_{m2}r_{o2}}{C}$$

Another limitation of such a system that performs in open loop is large gain variations, where even a small input voltage can saturate the system. Hence, the allowable input voltage range is basically defined by the transistors’ saturation voltage $\left(\pm 2\sqrt{V_{od}}\right)$, where the overdrive voltage (*V_od_*), $V_{od}=\sqrt{\frac{2I_{D}L}{K_{n}W}}$ plays an important role, as it can be a serious limitation for the maximum output current amplitude. To overcome this obstacle, either the bias current should be increased or the transistor sizes should be decreased. Increasing the bias current would significantly compromise the output resistance, since, as discussed earlier, the low frequency output resistance is defined by *r_o_*_4_, which should be in the range of MΩ to ensure accuracy. It is also known that *r_o_ =* 1/*λI_D_*, which dictates the fact that it would be a difficult task to achieve current in the range of mA. Considering the second option, which relates to reduction of transistors sizes, will result in small transconductance for input pairs. As mentioned earlier, although the introduction of a zero in the output impedance of the system improves its high frequency performance, the low frequency output impedance is degraded to some extent.

The addition of a large 100 pF on-chip capacitor resulted in a simulated output impedance of 2.5 MΩ at 1 MHz [13]. However, the low frequency output impedance was only 375 kΩ. Thus, an improvement is observed in terms of high frequency operation, with reduced accuracy in low frequencies.

The measurement results for loads from 100 Ω to 1 kΩ and operating frequency range of 10 kHz to 1 MHz provided 0.8% current variations where the maximum operating current was 500 µA_p-p_ [12]. The output impedance at 10 kHz was >1 MΩ, however, it was reduced to >160 kHz at 1 MΩ frequency. The THD of the maximum output current was 42 dB (0.79%), measured at 600 kHz [12]. The effect of stray capacitors, especially at the output, not only degraded the output impedance, but also produced significant phase shift, especially at high frequencies. The input/output phase delay was 3.6° at 1 MHz [12]. 

### 2.2. Closed Loop Linear Feedback Integrated Current Drivers

**Figure 7:**
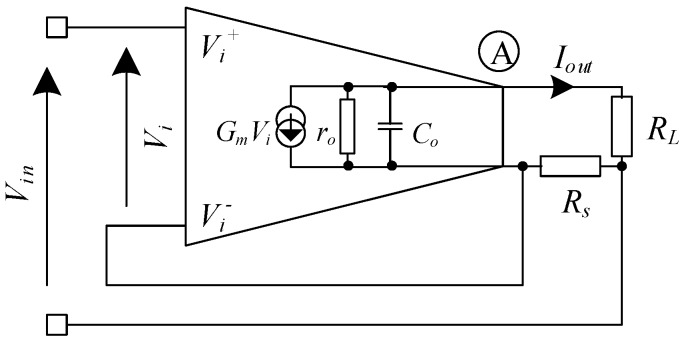
Basic linear feedback current driver [14].

The linear feedback current driver in [14] is based on a linear feedback system. The schematic is shown in Figure 7.

The current driver in this architecture employs a high transconductance OTA (*G*) in a negative feedback configuration to sense and regulate the current through the load. The generated output current flows through the load resistor (*R_L_*) that is in series with the sense resistor (*R_s_*). The resulting voltage controls the difference between *V_in_* and *V_i_*, thus monitoring and modifying changes in the output current. 

The output current of the driver is:

(11) $$I_{out}=\frac{V_{in}}{R_{s}}\frac{1}{1+\frac{1}{G_{m}R_{s}}\left(1+\frac{R_{L}+R_{s}}{r_{o}}\right)}$$

where (*G_m_*) is the transconductance, (*r_o_*) is the output resistance of the transconductor, (*R_L_*) is the load resistance, and (*R_s_*) is the sense resistance. Equation (11) illustrates the dependency of output current upon the OTA’s internal parameters (*G_m_*, *r_o_*), the load resistance (*R_L_*), and the sense resistance (*R_s_*). For *r_o_ >> R_L_ + R_s_* and *G_m_R_s_* >>1, the equation then reduces to

(12) $$G_{drive}=\frac{I_{out}}{V_{in}}=\frac{1}{R_{s}}$$

The overall transconductance of the circuit is only dependent on the passive component *R_s_*, which can be accurately trimmed in integrated circuits and is not a function of circuits internal parameters. 

The output impedance of the current driver is:

(13) $$R_{out}=r_{o}+\left(G_{m}r_{o}+1\right)R_{s}$$

If $G_{m}r_{o}>>1$ and $G_{m}r_{o}R_{s}>>1$

(14) $$R_{out}=G_{m}r_{o}R_{s}$$

As observed in Equation (14), the output resistance of the current driver is enhanced through the negative feedback configuration by multiplication of the OTA’s open loop gain factor (*G_m_r_o_*) with the sense resistor. Hence, the OTA’s output stage is simply increased without any design techniques to reach the output resistance in the MΩ region for accurate current measurements. The utilization of negative feedback increases the operating bandwidth of the driver. Amplifiers with high open loop gain are not crucial in the design. Nevertheless, this current driver suffers from two major drawbacks. 

The first problem arises due to voltage imbalance across load. This is the outcome of the voltage drop across the sense resistor as a result of output current passing through it [15]. This voltage imbalance results in common mode voltages that can be troublesome in bioimpedance measurements. Common mode voltages, due to imbalances, are at the same frequency as differential voltages, and their suppression is limited due to the degradation of the amplifier’s CMRR at higher frequencies [15]. The presence of common mode signals can result in differential signal measurement errors, which can lead to false impedance estimations. Finally, common mode voltage errors in bioimpedance measurements can sometimes be higher than differential voltages at the input of the measuring differential amplifiers due to the high input impedance [15].

Another disadvantage of this topology is the need to employ floating input voltages. The application of a floating input isolates the load from any direct path to instrument ground. In addition, it serves as a reference to the inverting terminal of the OTA when the voltage across the sense resistor changes [15]. The measured voltage developed across the sense resistor should provide a direct indication of the load current. Hence, shunting the load current will affect the accuracy in the regulation of the output current. Although transformer coupling can be utilized to generate balanced floating AC inputs, this is impractical to implement in CMOS. 

**Figure 8:**
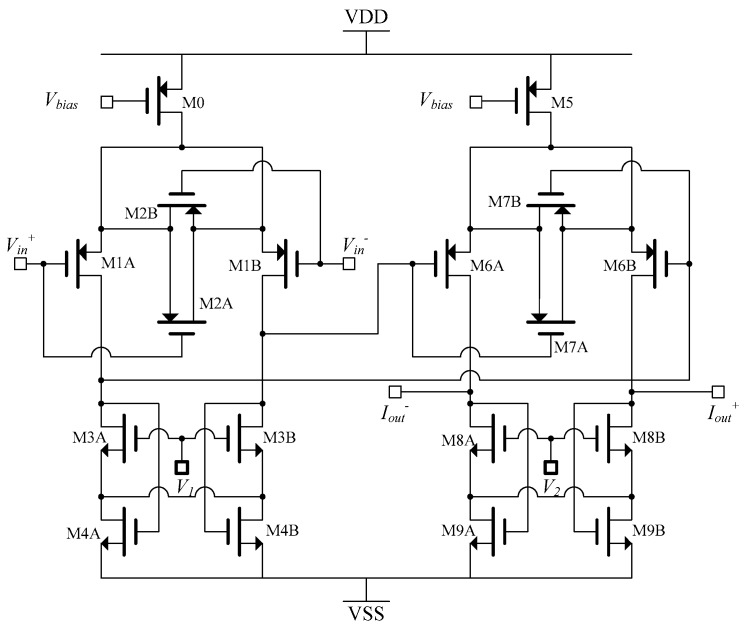
A high transconductance OTA [14].

The transconductor was implemented by using two cascaded OTAs to achieve large transconductance, as shown in Figure 8 [14].

Transistors M1A, M1B, M2A, and M2B form a source degenerated voltage to current converter. M3A and M3B operate as active loads, where M4A and M4B provide feedback to stabilize the common mode at the output. 

The overall transconductance of the OTA is given by:

(15) $$G_{m}=G_{m1}r_{o1}G_{m2}$$

where *G_m_*_2_ (= *G_m_*_1_), is the transconductance of the second OTA.

The simulation result shows an output impedance of 10 MΩ up to 10 kHz, which degraded to 1 MΩ at 1 MHz frequency with the presence of a 1 kΩ load [14]. With a supply voltage of ±2.5 V, the drive current capability is up to 500 μA_p-p_. The THD for the maximum current is –67 dB at 10 kHz and degrades to −52 dB at 1 MHz [13].

When frequency effects are considered, the transconductance (*G*) normally has two poles, one of which is dominant to ensure stability. Although the finite loop gain starts to roll off at cut-off frequency and degrade the output resistance to some extent, the effect of finite bandwidth can be modeled as the output resistance *R_out_* in parallel to a capacitor *C_out_*.

Therefore, the open loop frequency response is given by:

(16) $$A_{ol}=\frac{A_{ol}}{1+j\omega \frac{\omega }{\omega_{c}}}$$

where *ω* = 1/*R_out_C_out_*. Considering Equation (14), due to low frequency dominant pole *ω_d_* in the two-pole loop gain, the output impedance at high frequencies is not resistive, but approximately capacitive. This capacitor is [16]:

(17) $$C_{out}=\frac{1}{\omega_{d}R_{s}G_{m}r_{o}}$$

The value for this capacitor is in order of 2–10 pF, in addition to any added parasitic capacitance. The output impedance is frequency dependent, and at high frequencies it decreases, which results in degraded current accuracy. Moreover, due to the capacitor, the output phase delay increases at higher frequencies and creates phase delay between the input and output of the current driver. 

There can be a large offset at the output of the high transconductance OTA in Figure 8, mainly due to the fact that the output DC voltage of each OTA is controlled through the quiescent output resistance of the triode transistors of the corresponding transconductor, provided as common mode feedback for differential output. This quiescent resistance is dependent upon device dimensions. As a result, in the presence of any fabrication inaccuracies or device mismatches, the output DC level cannot be accurately defined, and results in large DC offset.

**Figure 9:**
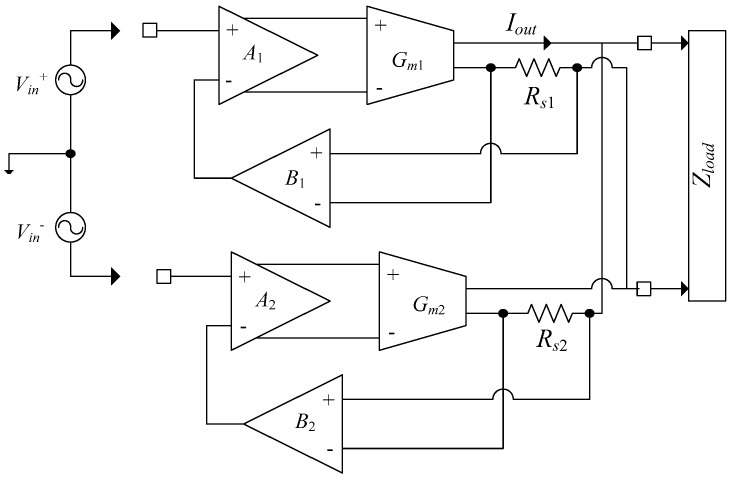
High power current driver [17].

The current driver in [17] has been introduced to overcome the two major limitations of floating input and voltage imbalance at the load in [14]. The block diagram of the topology with two identical differential feedback current drivers is shown in Figure 9.

The use of two identical differential feedback current drivers operating in a balanced mode minimizes common mode voltage errors across the load (*Z_load_*). The difficulty about floating inputs is eliminated by utilization of the two voltage buffers (*B*_1_*, B*_2_) in the feedback loop, isolating the load from the input signal. 

As discussed in [17], each driver is composed of a preamplifier stage, followed by a transconductance stage. Current through the load is sensed via sense resistors where the resulting voltage is fed back to the negative terminal of the respective preamplifier, thus establishing a negative feedback loop. The transconductance of the circuit is [17]:

(18) $$G_{drive}=\frac{I_{out}}{V_{in}}=\frac{1}{R_{s}+\left(\frac{r_{o}+R_{s}+R_{L}}{r_{o}}\right)\frac{1}{AG_{m}}}$$

where *I_out_* is the output current of the driver, *V_in_* is the input voltage, *R_s_* is the sense resistor, *A* is the gain of the preamplifier stage, *G_m_* is the small signal gain, and *r_o_* is the small signal output resistance of the transconductance stage. 

If $r_{o}>>R_{s}+R_{L}$ and $AG_{m}R_{s}>>1$, then

(19) $$G_{drive}=\frac{1}{R_{s}}$$

As a result, the transconductance of the current drive is only dependent on the sense resistor, not the circuit’s internal parameters. The total transconductance is twice the transconductance of a single drive, but the total output impedance is halved, since the two current drivers are in parallel [17]. The output impedance is enhanced through the negative feedback as follows:

(20) $$R_{out}=r_{o}+\left(AG_{m}r_{o}+1\right)R_{s}$$

In the frequency range of 10 kHz–500 kHz the accuracy of the output current is 0.41% for maximum current of 2.5 mA. For 10 kHz–1 MHz the accuracy for the maximum current is 0.47% [17]. The measured output impedance is 665 kΩ at 100 kHz and 64 kΩ at 1 MHz. The phase delay increased as the frequency was increased. In [17], the circuit can deliver a maximum output current of 5 mA_p-p_ with an accuracy of 0.41% in the frequency range up to 500 kHz, together with enhanced output voltage compliance of 15 V. A THD of less than 1% is achieved for an output current of 5 mA_p-p_. At 1 MHz frequency the phase delay was 12°.

A major drawback of this system is its stability. Sharp peaking in the frequency response of the system is the result of insufficient phase margins as the frequency approaches its upper limit. This excess phase delay, which is the result of two closely placed poles of transconductor and buffer, is compensated by a 60 pF on-chip capacitor for each driver. The compensation capacitor at the output of the preamplifier stage moves the gain crossover point more towards the origin. The gain-crossing point reaches unity well before the phase crossing point reaches −180° and provides enough phase margin for stability. Although this approach retains the gain and output swing, it reduces the bandwidth [18]. 

Considering the frequency effects in the two pole loop gain system (*AG_m_r_o_R_s_*) according to (17), due to presence of low frequency dominant pole (*ω_d_*) of the system, the output impedance at high frequencies is not resistive but approximately acts as a capacitor [16]. Therefore, as the frequency is increased, the phase delay introduced as the result of the capacitive impedance increases. This phase delay between the input and output directly affects any impedance measurement accuracy. 

Another limitation of this design is the increased power consumption required in comparison to other CMOS designs, as using two current driver circuits operating in parallel for the purpose of load voltage balancing doubled the total power consumption.

Although the model for two parallel current drivers was established for balancing the output voltage, in reality, some DC offset is present at the output. This is mainly due to the fact that the output DC voltage of each driver is controlled through the quiescent output resistance of the triode transistors of the corresponding transconductor provided as common mode feedback for differential output transconductor. This quiescent resistance is dependent upon device dimensions. As a result, in the presence of any fabrication inaccuracies or device mismatches, the output DC level cannot be accurately defined and results in a large DC offset.

**Figure 10:**
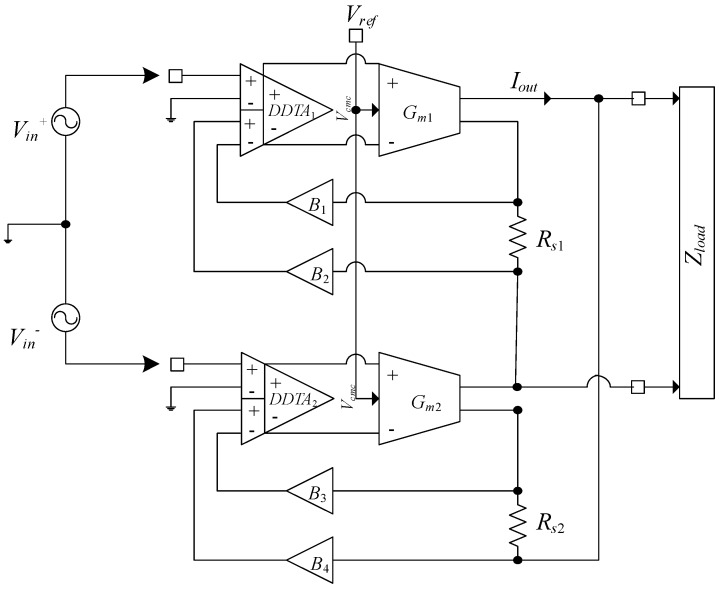
Alternative current driver topology (optimized for supply voltage of ±2.5V) [19].

In [19], a similar architecture has been used in a 0.35 μm standard CMOS technology that operates with a ±2.5 V supply. The schematic of the current driver is shown in Figure 10.

The circuit employs a pre-amplification stage by utilizing a differential difference transconductance amplifier (DDTA_1_*,* DDTA_2_) followed by a transconductance stage (*Gm*_1_, *Gm*_2_) performing the voltage to current conversion. The output current is sensed by two on-chip resistors (*R_s_*_1_*, R_s_*_2_), whose voltage is fed back via four single to single-ended buffers (*B*_1_–*B*_4_) into the negative terminal of the preamplifier, thus establishing a negative feedback loop [19]. 

The output compliance of the current driver is 4 V, with a maximum output current capability of 1 mA_p-p_. To overcome the DC offset issue in the previous design, an auxiliary common mode voltage at the input of the transconductor is defined. The current driver can deliver a maximum output current of 500 μA_p_ with an accuracy of 0.44% in the frequency range of up to 800 kHz, and a THD of less than 0.26% was achieved for an output current of 1 mA_p-p_ [19]. The output impedance is 1 MΩ at 500 kHz, and approximately 360 kΩ at 1 MHz. The input/output phase delay at 1 MHz is 9.5° [19]. Although performance was improved for low power consumption, the issue of instability is still present, which necessitates a large on-chip compensation capacitor. Moreover, due to the presence of a dominant pole at high frequency, which results in the output impedance acting as a capacitor rather than resistor, the generated phase error must be minimized by an added compensation scheme to reduce the effect of the capacitance at the output.

**Figure 11:**
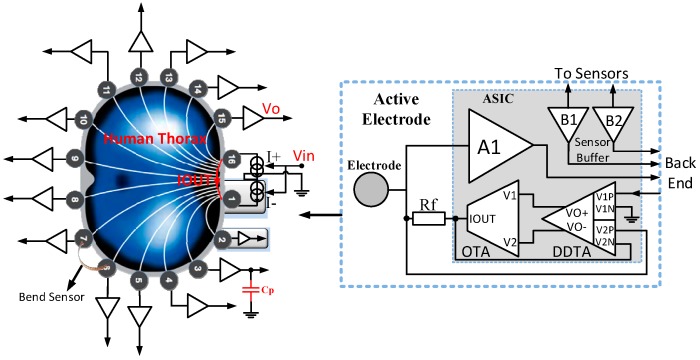

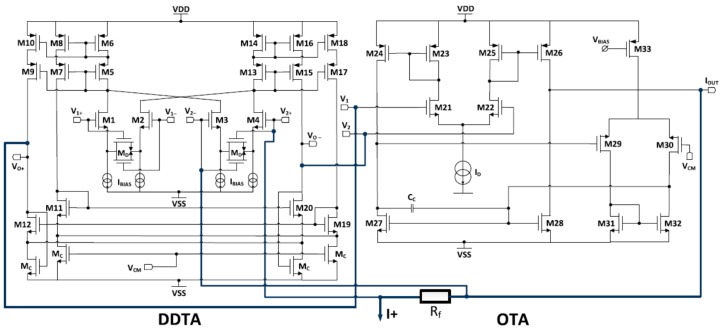
System level architecture and circuit level design of the current driver of an active electrode IC for wearable Electrical Impedance Tomography (EIT) (Reproduced from [20,21], with permission from the IEEE).

The proposed current driver in [20,21] is part of an active electrode IC for wearable Electrical Impedance Tomography (EIT). The current driver in Figure 11 has been designed in a 0.35 μm CMOS technology. It operates from ±9 V power supplies and occupies a total die area of 5 mm^2^.

The current driver is composed of a differential difference transconductance amplifier (DDTA), followed by a wideband operational transconductance amplifier (OTA). In this manner, the DDTA measures the output current through the sensor resistor, *R_f_*, and forms a linear feedback. In this EIT system, one current driver is configured as a current source and the other as a current sink, both working together to form a complete current path. The transconductance and output impedance are given respectively by:

(21) $$G_{m}=\frac{I_{out}}{V_{in}}=\frac{A_{loop}}{R_{f}+R_{load}+A_{loop}R_{f}}\approx \frac{1}{R_{f}}$$

where *A_loop_* is the overall open loop gain of the current driver and *R_load_* is the load.

(22) $$Z_{o}=r_{oOTA}+R_{f}\left[A_{DDTA}G_{mOTA}r_{oOTA}+1\right]$$

where *r_oOTA_* is the output impedance *I_out_* and *A_DDTA_* is the open loop gain of the DDTA.

The DDTA circuit compares two differential input signals (*V*1, *V*2). In this design, a fully differential output is provided by adding a duplicate of the output branch so that it can be connected to the wideband OTA [20]. 

Four source-degenerating transistors M_D_ are added to the cross-coupled input pairs of the DDTA to achieve a high output current amplitude with low distortion. Using the four triode–transistors M_C_ for common mode feedback, a bias voltage VCM can set the common mode voltage at the circuit’s output.

In the design, the OTA, a DC biasing stage (comprising M29 to M33), is configured to a feedback loop with M24 and M27 to provide a biasing voltage for transistors M27 and M28, and to set the DC level at *I_out_* [21]. 

In this system two single-ended current drivers have been utilized to provide source and sink currents for the corresponding electrodes, while a differential current driver with common mode feedback (CMFB) could be used to reduce the overall power consumption and size of the system. Any mismatch between the source and the sink currents would force the unmatched current to flow through the output impedance node of the current drivers, which results in a large common mode signal that can cause measurement errors, or even saturate the driver output.

**Figure 12:**
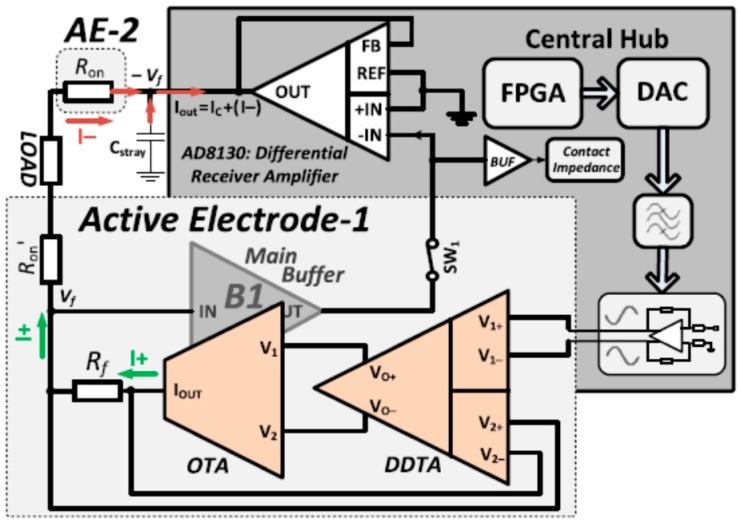
Current driver topology with sink current provided from central hub (Reproduced from [21] with permission from the IEEE).

In an analogous design in [21], a fully differential current driver is proposed, as shown in Figure 12. In *Active Electrode-1*, the source current is configured as in [20], while the sink current is provided from the central hub. A fully differential voltage signal is generated from the central hub as input to the current driver. The current driver sources a current of *I+* and the main buffer *B1* senses the voltage *Vf* directly on the load and feeds it back to the central hub, while the differential receiver amplifier senses *Vf* and generates an output voltage, which provides a voltage to generate sink current *I–* [21].

In this topology, the feedback amplifier generating the sink current is a negative feedback voltage amplifier which, by sensing the common mode signal, generates a voltage at its output which results in a sink current that matches the source current. Providing the voltage gain of the amplifier is high enough, the common mode is removed.

The output phase error of 4° at 500 kHz is not a good comparison with respect to other systems, where no phase errors were achieved at such frequency. Meanwhile, having the sense resistor off-chip provides more stable results and much less process variation as opposed to on-chip sense resistor, where the transconductance of the system is inversely proportional to its value. The current driver has a bandwidth of 500 kHz. The output impedance is 1.12 MΩ at 500 kHz. The maximum output current is 6 mA [21]. The design takes advantage of the closed loop architecture in terms of bandwidth and stability.

**Figure 13:**
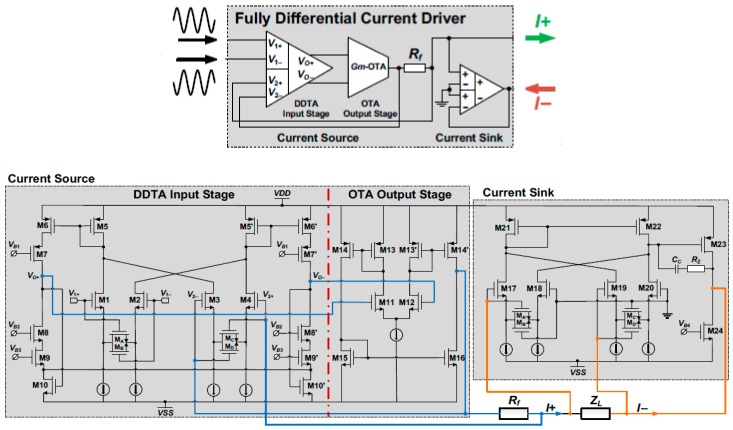
Circuit architecture of the current driver with common mode feedback (Reproduced from [23], with permission from the IEEE).

The current driver in [22] is an enhanced version of [21] for wearable EIT systems where a built-in common mode signal reduction is added. The driver chip is fabricated in 0.18 µm CMOS technology and operates with ± 1.65 V supply, with total die area of 0.05 mm^2^. Figure 13 shows the block diagram of the proposed current driver along with circuit level design. As illustrated in Figure 13, the master current driver consists of a DDTA followed by an OTA to enhance the overall transconductance. The slave differential voltage receiver (DVR) measures the voltage across the load and, through feedback, forces the common-mode voltage across the load to be zero [22]. Therefore, it sinks the current sourced by the master driver to form a complete current path. 

The transconductance of the current driver is:

(23) $$G_{m}=\frac{A_{olDDTA}G_{mOTA}}{1+A_{olDDTA}G_{mOTA}R_{f}}$$

The output impedance is:

(24) $$R_{o}=r_{oOTA}\left(A_{olDDTA}G_{mOTA}\right)R_{f}$$

The measurement results of the current driver chip show an output impedance of 750 kΩ at 500 kHz with maximum output current of 1mA_p-p_ and THD of 42 dB [22]. There is no information provided about any potential phase error. As explained earlier in previous current drivers, a higher bandwidth is not easily achieved, as the capacitor effects in higher frequency results in lower output impedance, degraded current accuracy, and phase errors.

### 2.3. Closed Loop Non-Linear Feedback Integrated Current Drivers

**Figure 14:**
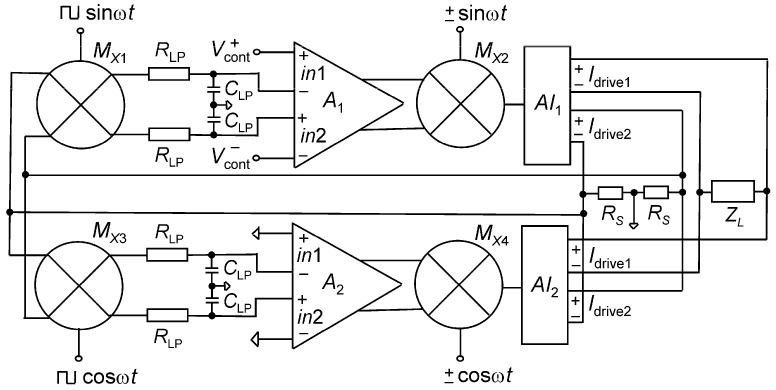
Block diagram of the current driver with its corresponding phase compensation. The upper part represents the current driver, where the lower part is the compensation with zero control voltage and *cosωt* (Reproduced from [25], with permission from the IEEE).

The current driver in [24] is based on non-linear feedback, as illustrated in Figure 14. The system has been designed in a 0.35 µm CMOS technology with ±2.5 V supply voltages.

The system has the advantage of the dominant pole not affecting its high frequency operation, as opposed to other linear feedback current drivers discussed earlier. However, the transient response is longer due to low frequency dominant pole of the lowpass filters [24].

The amplitude of the differential current was set by differential DC voltages of ±*V_cont_* and the frequency of operation was dictated by the frequency of differential *sinωt* and *cosωt* input signals at of multipliers *M_X_*_2_ and *M_X_*_4_, and the corresponding square waves at *M_X_*_1_ and *M_X_*_3_. The differential voltage across *R_s_* was converted to DC voltage through the switch multipliers *M_X_*_1_ and *M_X_*_3_ and four RC low pass filters (LPF) to extract the DC component [25]. It resulted in an output voltage of (2/π) *V_p_*/2 where *V_p_* was the peak voltage of the AC signal across *R_s_*. The resulting DC voltages were compared to differential control voltages and amplified by *A*_1_ in the current driver and compared to zero and amplified by *A*_2_ in the compensation circuit. The signal was then modulated back to AC via analogue multipliers *M_X_*_2_ and *M_X_*_4_ and current amplifiers *AI*_1_ and *AI*_2_. In the compensation circuit, the same circuit as the current driver an at 90^o^ phase shift with respect to main driver at *cosωt*, where (ω) is the desired frequency of operation of the current driver [24], which considerably reduces the phase delay of *I_drive_* to beyond the pole frequencies of *M_X_*_2_, *M_X4_*. The compensation circuit (lower circuit) senses the amplitude of the out-of-phase component of the voltage across *R_s_* and generates an amplified version of it. The outputs of both circuits were added and feedback nearly canceled out the out-of-phase components [24]. The detailed analysis of the phase compensation is provided in [16]. In this system configuration, there are two differential outputs of the multipliers *M_X_*_2_ and *M_X_*_4_: one drives the load *Z_L_* and the other is used for feedback via *R_s_*. 

As the presence of *R_s_* does not enhance the output resistance of the system, the high output resistance was attained by using high swing cascodes. On the other hand, since the dominant pole is placed at very low frequencies, the capacitor effects at higher frequencies are not a limiting factor in the output impedance measurements, and the system achieves an output of 1 MΩ over the entire bandwidth of operation [26]. The loop gain of the current driver is analogous to the linear feedback system [24] with an additional 2/π factor as a result of AC–DC conversion at the LPF. 

If *AGRs*>>1 then

(25) $$I_{drive\left(peak\right)}=\frac{\pi V_{cont.}}{R_{s}}$$

where *A* is the voltage gain of amplifiers *A*_1,2_, *G* is the transconductance gain of multipliers *M*_x2,4_ and current amplifiers *AI*_1,2_, and *R_s_* is the sense resistance.

The measurement results of the current driver shows the current amplitude without compensation varies by 1.8% for 1mA_p-p_, while the phase error starts to increase from 500 kHz and reaches 20° at 3 MHz [25,26]. With compensation, the maximum output of 1 mA_p-p_ has an accuracy of 0.24% and 1.3% at 1 and 2.5 MHz, respectively. With compensation, the phase error reduces to 1° at 1 MHz and 3° at 3MHz, regardless of the current amplitude [25]. The measured THD of the current driver for a current of 1 mA_p-p_ was 0.25% at 200 kHz, rising to 0.4% at 500 kHz.

Although the system provides superior results in terms of output impedance, phase error, and bandwidth with respect to other linear systems, the design is more challenging and occupies more area.

## 3. Conclusions

As a main building block in the design of any bioimpedance measurement system, a current driver plays a vital role. A comprehensive study on the state-of-the-art integrated current drivers has been provided in this paper. Transconductance, output impedance, current amplitude, and phase error, along with other aspects, have been discussed in each system. Table 1. provides a brief comparison of these current drivers, mostly based on the measurement results.

## Figures and Tables

**Table 1:** Comparison of measurement (otherwise stated) results for various integrated current drivers.

Ref.	Output Current	Bandwidth	Output Impedance	Phase Error	THD
[5]	107 µA_p-p_ (simulation)	10 MHz (simulation)	10.2 MΩ at 1 MHz (simulation)	-	1% 10 MHz (simulation)
[7]	100–350 µA_p-p_	90 kHz	560 kΩ at 90 kHz	-	1% for 250 µA_p-p_
[8]	80, 200 and 400 µA_p-p_	10–76 kHz	-	-	<0.2% for 200 µA_p-p_
[9]	0–169 µA_p_	-	-	-	-
[10]	7 µA_p_	1, 8 and 16 kHz	-	-	-
[11]	16–512 µA_p_	DC-500 Hz	1.6 GΩ	-	-
[12]	500 µA_p-p_	10 k–1 MHz	>160 kΩ at 1 MHz	3.6° at 1 MHz	0.79% for 500 µA_p-p_
[14]	500 µA_p-p_ (simulation)	1 MHz (simulation)	1 MΩ at 1 MHz (simulation)	-	−52 dB at 1 MHz (simulation)
[17]	5 mA_p-p_	1 MHz	64 k kΩ at 1 MHz	12° at 1 MHz	<1% for 5 mA_p-p_
[19]	1 mA_p-p_	1 MHz	360 kΩ at 1 MHz	9.5° at 1 MHz	0.26% for 1 mA_p-p_
[20,21]	6 mA_p-p_	500 kHz	1.12 MΩ at 500 kHz	4° at 500 kHz	55 dB
[22,23]	1 mA_p-p_	500 kHz	750 kΩ at 500 kHz	-	42 dB for 1 mA_p-p_
[25,26]	1.6 mA_p-p_	100 kHz – 3 MHz	> 1MΩ at 3 MHz	3° at 3 MHz	0.4 % for 1mA_p-p_
